# Improving access to highly effective emergency contraception: an assessment of barriers and facilitators to integrating the levonorgestrel IUD as emergency contraception using two applications of the Consolidated Framework for Implementation Research

**DOI:** 10.1186/s43058-022-00377-0

**Published:** 2022-12-09

**Authors:** Rebecca G. Simmons, Jami Baayd, Sarah Elliott, Susanna R. Cohen, David K. Turok

**Affiliations:** 1 Division of Family Planning, Department of Obstetrics and Gynecology, University of Utah, 30 North 1900 East, Salt Lake City, UT 84132 USA; 2 LIFT Simulation Design Lab, Division of Family Planning, Department of Obstetrics and Gynecology, University of Utah, 30 North 1900 East, Salt Lake City, UT 84132 USA

**Keywords:** Contraception, Emergency contraception, LNG IUD, Intrauterine device, CFIR, Implementation, Simulation

## Abstract

**Background:**

Emergency contraception prevents unwanted pregnancy after sexual intercourse. New evidence has demonstrated that the levonorgestrel 52 mg IUD is a highly effective method of emergency contraception. However, translating this research finding into clinical practice faces existing barriers to IUD access, including costs and provider training, novel barriers of providing IUDs for emergency contraception at unscheduled appointments. The purpose of this study was to identify barriers and facilitators to the utilization of the levonorgestrel IUD as emergency contraception from client, provider, and health systems perspectives.

**Methods:**

We conducted English and Spanish-speaking focus groups (*n*=5) of both contraceptive users (*n*=22) and providers (*n*=13) to examine how the levonorgestrel IUD as EC was perceived and understood by these populations and to determine barriers and facilitators of utilization. We used findings from our focus groups to design a high-fidelity in-situ simulation scenario around EC that we pilot tested with clinical teams in three settings (a county health department, a community clinic, and a midwifery clinic), to further explore structural and health systems barriers to care. Simulation scenarios examined health system barriers to the provision of the levonorgestrel IUD as EC. We coded both focus groups and in-clinic simulations using the modified Consolidated Framework for Implementation Research (CFIR). We then applied our findings to the CFIR-Expert Recommendations for Implementing Change (ERIC) Barrier Busting Tool and mapped results to implement recommendations provided by participants.

**Results:**

Ultimately, 9 constructs from the CFIR were consistently identified across focus groups and simulations. Main barriers included suboptimal knowledge and acceptability of the intervention itself, appropriately addressing knowledge and education needs among both providers and contraceptive clients, and adequately accounting for structural barriers inherent in the health system. The CFIR-ERIC Barrier Busting Tool identified eight strategies to improve levonorgestrel IUD as EC access: identifying implementation champions, conducting educational meetings, preparing educational toolkits, involving patients and their partners in implementation, conducting a local needs assessment, distributing educational materials, and obtaining patient feedback.

**Conclusions:**

To sustainably incorporate the levonorgestrel IUD as EC into clinical practice, education, health systems strengthening, and policy changes will be necessary.

Contributions to the literature

- Emergency contraception is an important means of preventing unintended pregnancy. Recent evidence supports the use of a new option, the levonorgestrel 52 mg IUD, as a form of emergency contraception.
- Through focus groups and simulations we identified individual, provider, and clinic-level barriers to the use of levonorgestrel IUDs for emergency contraception.
- We used well-established implementation frameworks to pair the identified barriers with specific recommendations for how to overcome those barriers.
- We utilized a simulation scenario to further identify previously unidentified barriers and facilitators in clinical settings

## Background

Emergency contraception (EC) is a critical tool to reduce unwanted pregnancy after unprotected intercourse. Until recently, there were three options for emergency contraception in the United States: oral levonorgestrel (LNG), oral ulipristal acetate, and the copper IUD. Each of these methods has benefits and limitations in their use (see Table 1).

**Table 1:** Overview of available methods of emergency contraception

Method	Rules for use	Mechanism of action	Efficacy	Availability	Accessibility
**Copper IUD**	Placement within 120 h after unprotected intercourse (UPI)	Disruption of sperm and ovum function; possible interference with implantation	<0.01 pregnancies per single use cycle 0.06 pregnancies for 10 years after insertion	In-clinic insertion via healthcare provider	Free under the Affordable Care Act; >$1000 if paying out-of-pocket
**Levonorgestrel IUD**	Placement within 120 h after UPI	Interferes with sperm transport, capacitation	<0.03 pregnancies per single use cycle 0.07 pregnancies for 5 years^b^ after insertion	In-clinic insertion via healthcare provider	Free under the Affordable Care Act; Between $100->1000^a^, if paying out-of-pocket
**Oral levonorgestrel**	Take as soon as possible within 120 h after UPI (^a^most effective within 72 h)	Delays ovulation	1.7–2.6 pregnancies per single-use cycle	Over-the-counter	$5–$75 per pill
**Ulipristal acetate**	Take within 120 h after UPI	Delays ovulation	1.2–1.8 pregnancies per single-use cycle	Prescription-only	Free under the Affordable Care Act; Between $35–75, if paying out-of-pocket

*IUD* intrauterine device, *UPI* unprotected intercourse

^a^Cost is variable and dependent on both the IUD type and clinic participation in other programs, such as 340b pricing

^b^ Duration of use is dependent on the type of LNG IUD used; 52 mg IUDs can last up to 7 years after insertion

For example, oral LNG is available over-the-counter, making it the most widely used method of emergency contraception in the United States, but this has also resulted in it not being billable to insurers. With widely variable pricing and the need to pay out-of-pocket, it is often cost-prohibitive to people with lower incomes. Among these commonly used EC methods, oral levonorgestrel is the least effective (pregnancy rates of 1.7–2.6 per single cycle of use) [1–3], is less forgiving of use beyond 72 h after unprotected intercourse, and its efficacy is further limited by increasing body weight. A recent study found that doubling the dosage of oral levonorgestrel did not improve rates of unintended pregnancy among people at higher body mass index (BMI) [4].

Ulipristal acetate is the most effective oral emergency contraceptive (pregnancy rates of 1.2–1.8 per single cycle of use [2, 3, 5]) with a wider dosing window (120 h after unprotected intercourse) and is effective for people at higher BMI, but is only available by prescription. As such, barriers to its timely access (needing to first meet with a healthcare provider and then have a prescription filled at the pharmacy, which often does not stock ulipristal acetate and thus must order it — resulting in increased delays) are much higher, resulting in very low utilization of this method.

The copper intrauterine device (IUD) is the most effective of the available methods at preventing pregnancy (<0.1% of use results in pregnancy) and provides a long-term solution to prevent pregnancy after use [6]. However, among IUDs, the copper IUD is less popular than the LNG IUD because of some of the side effects associated with the copper IUD, such as a heavier bleeding profile [3, 7, 8]. For ongoing contraception, many people prefer the LNG IUD because it reliably reduces or eliminates menstrual bleeding and discomfort [9–11]. Despite the preference shown to LNG IUD over the copper IUD, until recently patients have not been able to receive the LNG IUD for emergency contraception, due to a lack of sufficient efficacy data [12]. A recently conducted randomized controlled trial found that the LNG 52 mg IUD (please note all mentions of LNG IUD in this paper refer to the 52mg variety) demonstrates high efficacy for emergency contraception [13]. The participant-blinded randomized noninferiority trial compared outcomes of women seeking an IUD as EC who received either the 52 mg LNG IUD or the copper IUD. One-month pregnancy rates were 0.3% (95% CI: 0.1, 1.7) in the LNG group and 0.0% (95% CI: 0, 1.1) in the copper IUD group, demonstrating that both methods are effective in preventing pregnancy when used as emergency contraception [13]. This efficacy data opens the door for LNG 52 mg IUDs to be the next method option for emergency contraception: the first new method of emergency contraception since ulipristal acetate was FDA-approved for oral emergency contraception in 2010 [3]. Now is a critical time to implement these findings because both emergency contraception and IUD use are steadily increasing, with over one fourth of reproductive age women reporting having used emergency contraception and greater than 1 in 10 contraceptive users selecting IUDs.

If the LNG IUD were broadly available as emergency contraception, it may prove to be the more preferred IUD emergency contraception method and offer additional benefits to people seeking an IUD for their emergency contraception needs. Studies have demonstrated that offering a wider selection of methods increases contraceptive satisfaction and reduces unintended pregnancy [14]. Expanding method choice for emergency contraception will have an extensive clinical impact if these findings can be broadly disseminated and implementation barriers can be identified and addressed early.

To date, dissemination and implementation of best practices surrounding contraceptive research is limited [15]. Successful translation of research typically takes many years and may be stymied by unforeseen or unaddressed barriers to implementation [16]. For example, while IUDs and implants are in high demand, access barriers such as lack of provider training on insertion and removal still impede their wider availability, particularly in primary care settings [17]. Uptake of the LNG IUD as emergency contraception will face some of those same existing barriers, as well as additional challenges to use, such as the need to provide same-day services [18]. Additionally, providers will need to be educated on how to counsel on IUDs as a method of emergency contraception, and patients will need to know that IUDs are an option for EC. To ensure the clinical research regarding the effectiveness of LNG IUDs as EC is translated into practice, the next critical step is to assess possible barriers and facilitators to use of LNG IUDs as EC in a clinical setting.

This study sought to address the issue of successful implementation by collecting and analyzing data on key aspects of patient, provider, and health system barriers and facilitators to implementing the LNG IUD as emergency contraception, as well as identify potential implementation strategies for future interventions. The barriers and facilitators combined with solutions identified in this study should help to develop guidance and recommendations for best practice to implement IUD as emergency contraception as well as health systems strengthening mechanisms to support clinics, providers, and patients facing barriers to emergency contraception access.

### Methods

To understand the barriers and facilitators to provide LNG IUDs as emergency contraception, we conducted exploratory research in three ways: (1) focus groups with clinical providers who offer contraceptive care in practice, including IUDs; (2) focus groups with community members who have had prior experience with contraceptive care (any method); and (3) in-clinic simulation training including providers and clinical staff. The focus groups allowed us to investigate facilitators and barriers from both the patient and provider perspectives. The simulations allowed us to expand from individual perspectives to health system-level barriers, including organizational and workflow limitations.

### Focus groups

We developed semi-structured discussion guides for both provider and community member focus groups. The discussion guides were built around key constructs from the Consolidated Framework for Implementation Research (CFIR) [19], an implementation tool which was also used to guide our analysis. The provider discussion guide focused on providers’ clinical knowledge of IUDs, their understanding and experience of using IUDs as EC, as well as examining how providers typically receive updated clinical guidance and care recommendations. The community member discussion guide focused on understanding participants’ experiences with IUDs (particularly hormonal IUDs) and capturing their knowledge and beliefs around the use of IUDs as emergency contraception.

Community member participants were recruited from the HER Salt Lake research study, a prospective cohort study that occurred between September 2015 and March 2017. The HER Salt Lake sample consisted of women aged 18–45 years of age receiving new contraceptive services at health centers in Salt Lake County, Utah [10]. We only contacted participants who indicated on prior consents that they were willing to participate in future research, were of reproductive age (between 18-45), were current or prior residents of Utah, and were currently trying to prevent pregnancy. Additionally, we recruited participants through University of Utah-affiliated community Latine/a/o organizations. Participants were consented and included on a first response basis up to 20 participants, per group, to account for scheduling conflicts and unexpected no-show participants during the focus group. English-speaking focus groups were conducted by members of the study team (RS and SE) and the Spanish-speaking focus group was conducted by a local community facilitator fluent in Spanish. All interviewers were female, held higher education credentials, and had prior experience and training in conducting focus groups. Focus groups occurred and were recorded on Zoom. All focus groups took approximately 60 min.

Healthcare providers were recruited by contacting community clinicians participating in Family Planning Elevated, a Utah statewide contraceptive initiative [20], as well as University of Utah faculty listservs to women’s health care departments. Providers were eligible to participate if they were currently employed as a healthcare clinician and currently offering contraceptive care as part of their healthcare practice. All provider types (e.g., physician, physician’s assistant, nurse practitioner) meeting these criteria were considered eligible for these focus groups. Focus groups occurred and were recorded on Zoom. All focus groups took approximately 60 min.

Once a potential participant expressed interest, an enrollment email was sent with the full consent language both as an attachment and in the body text of the email. Participants were enrolled and included in the focus group if they responded affirmatively to the consent communication. Participants received gift card compensation for their time. All focus groups were audio recorded and transcribed verbatim. The Spanish-speaking focus group audio was translated by the University of Utah language translators, who provide all professional health language translation for the university.

### Simulation scenarios

**Fig. 1:**
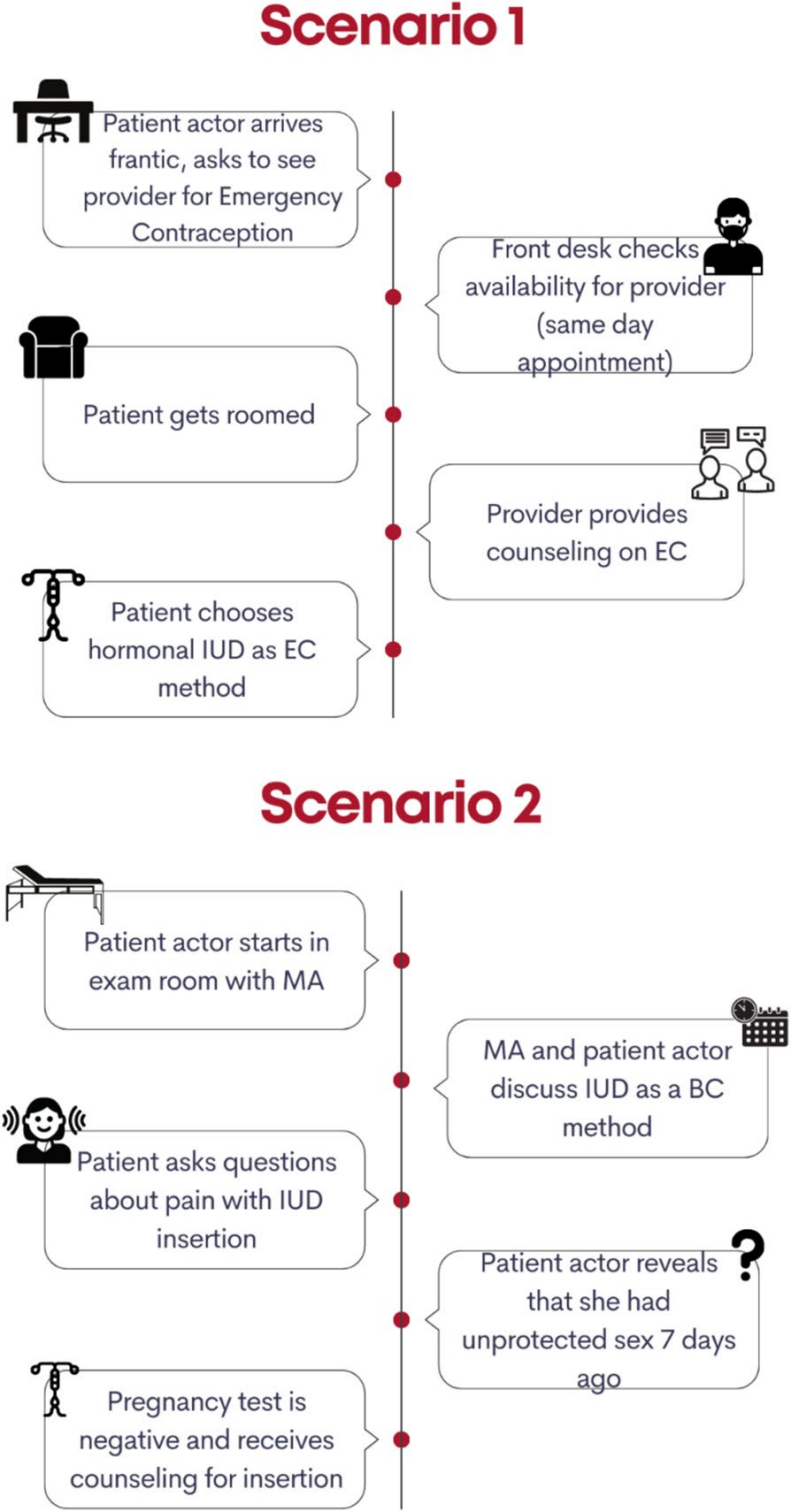
Simulation scenario

The LIFT Simulation Design Lab, at the University of Utah, designed a 2-h emergency contraception clinical training. Simulation was selected as an appropriate method to gain insight within a clinical setting as there is a significant body of evidence from varied clinical settings that demonstrates the value of incorporating highly realistic simulation techniques into in-service training for improving clinical decision-making, teamwork, and use of evidence-based practices [21–24]. Simulation can both identify barriers and facilitators of health systems implementation, and provide opportunities for technical education and improvement to team communication [25–28]. The simulation training was designed with the following components: (1) brief didactic training sharing the current evidence around the efficacy of the LNG 52 mg IUD as EC, as well as a review of currently offered methods of EC; (2) simulations scenario(s) with facilitated debrief; and (3) discussion of barriers/facilitators to EC access in the clinical setting (see Fig. 1).

The simulation training was pilot tested within the University of Utah’s Family Planning Division team prior to clinic recruitment. We recruited clinics via email, inviting them to participate in a 2-h in-clinic simulation training on the use of LNG IUDs as EC. Clinics recruited included those which had participated in Family Planning Elevated and clinics which had expressed prior interest in engaging around contraceptive training. Clinics were eligible to participate if IUDs were offered as a contraceptive method at the time of the simulation. Participants were consented prior to participation by emailing the consent document to participating staff. Prior to initiating the training, the consent was reviewed, and assent confirmed. Participating clinics did not receive financial compensation for participation.

The trainings were conducted collaboratively by the University of Utah Family Planning team and LIFT Lab simulation team members. Each of these individuals is female, possesses higher education credentials, and has had prior experience and training in conducting simulations in clinical settings. One member of the Family Planning team (JB) collected field notes during the training. The field notes included a list of barriers the clinic teams identified during the training, as well as any solutions they identified to those barriers. Following each training, those barriers and solutions were organized into memos containing key points, barriers, and solutions from each training.

### Analysis

The study follows the COnsolidated criteria for REporting Qualitative research (COREQ) guidelines for qualitative research (see Fig. 1). All focus group audio recordings were transcribed, verbatim, and the Spanish focus group was translated into English. Transcripts were uploaded to Dedoose Version 8.0.35 [29]. The research team conducted a content analysis of the data, using an adapted the CFIR codebook [19] for use in this study. CFIR supports rapid-cycle evaluation of the implementation of complex health care delivery interventions due to its comprehensive framework for identifying factors that may emerge in various, multi-level contexts that subsequently influence implementation. The initial codebook included 39 codes. The team collaboratively (RS, JB, SE) coded one provider focus group and one community member focus group, and further refined the CFIR codebook based on which codes emerged as salient, and which did not, until saturation occurred. The revised codebook contained 18 CFIR constructs. Final transcript coding was conducted by individual team members (JB, SE) using the refined codebook. Codes and representative quotes were organized into a CFIR matrix, following the principles of Framework Analysis [30]. Finally, field notes detailing clinic-level barriers from each of the simulation trainings were also mapped onto the CFIR matrix, with solutions categorized separately.

After completing coding and mapping onto the framework, we subsequently ran our findings through the CFIR-ERIC Barrier-Buster tool (V0.53) which was developed to match CFIR constructs to corresponding Expert Recommendations for Implementing Change (ERIC) strategies [31]. The top eight endorsed ERIC implementation strategies were compared and mapped along with proposed solutions provided by participants. We utilized the refined compilation of implementation strategies [32] to provide further clarifying language around recommended approaches.

## Results

Twenty-two individuals participated in the three client focus groups (6 in the Spanish-speaking group; 16 in the two English-speaking groups). The two provider focus groups consisted of 13 participants: four medical doctors, four certified nurse-midwives, four nurse practitioners, and one physician assistant. Seven providers are employed within the University of Utah and six are employed in community clinics within the state of Utah. All participants currently provide contraceptive care in the state of Utah.

Four clinics received the emergency contraception simulation training: one county health department, two community clinics, and one midwifery practice. Participating clinic staff included nurse practitioners, registered nurses, physician assistants, medical assistants, clinic managers, front desk managers, and nursing students.

Table 2 provides an overview of CFIR constructs identified through focus groups and simulations, with descriptive quotations for each construct. Of the original eighteen constructs in our revised CFIR codebook, nine constructs were used most frequently across the groups. We combined “Structural Characteristics” and “Complexity” into one construct, as they had considerable overlap in our results and have sufficient conceptual overlap as constructs to warrant combining. Structural characteristics describe the setting in which the intervention is implemented, and compatibility describes the degree of fit between the intervention and implementation site.

**Table 2:** Recommended strategies for implementing use of LNG IUD as EC

CFIR-ERIC Barrier Buster Tool Implementation Strategies Recommended^a^	Parallel strategies proposed by study participants	Mapping summary
**CFIR Construct:** Culture **ERIC recommendation: Identify and prepare champions-** Identify and prepare individuals who dedicate themselves to supporting, marketing, and driving through an implementation, overcoming indifference or resistance that the intervention may provoke in an organization	*“If ACOG or someone came out saying that it was appropriate and safe that people would feel a lot more easy… they would feel a lot more comfortable doing it simply from a CYA [cover your ass] perspective. I think people are always paranoid that they’re going to do something wrong and if it’s supported in guidelines I certainly imagine that more people would feel comfortable with it.”* *- Provider* *I think in our clinic just because sometimes I feel like we’re a little bit more under the microscope. We would probably just really consult with general OB with… I mean we’d probably talk to [Department Chair] and be like, “Listen, are you guys on board with this or not?” And if there's general consensus that everybody’s on board we would do it but if we didn’t get that sort of nod, we’d probably be like, “Yeah, not doing it.”* *- Provider*	Implementation champions should include both local individuals and those within governing/formal bodies
**CFIR Construct(s):** Access to Knowledge & Information/Knowledge & Beliefs about the Intervention **ERIC Recommendation: Conduct educational meetings -** Hold meetings targeted toward different stakeholder groups (*e.g.*, providers, administrators, other organizational stakeholders, and community, patient/consumer, and family stakeholders) to teach them about the clinical innovation	*“You also have, other providers have to do CME. If this is something that is introduced in some of the main conferences that people use for their continuing ed, that’d probably help.”* *- Provider* *“Giving informational lectures in churches works well like with mammograms. The church is where women find out where they can go, how, when, and at what cost.”* *- Client*	Identifying and using existing venues of knowledge distribution for both clinicians and clients could facilitate educational meetings.
**CFIR Construct:** Access to Knowledge & Information **ERIC Recommendation: Develop educational materials-** Develop and format manuals, toolkits, and other supporting materials in ways that make it easier for stakeholders to learn about the innovation and for clinicians to learn how to deliver the clinical innovation	*“I love the little laminated sheet that I don't know where it comes from. You guys could probably tell me but you guys have all seen. I have the [education materials] that shows your options and makes it so clear to the patient. Then the one that says — Oops, and then like tells you what the emergency contraception options are. [. . .] if you could replicate those somehow and then make one that includes the LNG IUD, that would be helpful and a good reminder to both patients and providers.”* *-Provider* *Just something along the lines of like the bathroom stall flyers to get hung. . .. Just — here are your emergency contraceptive options. By the way, there's a long-term contraceptive option that can be also used as emergency contraception so you could get both things at once. . . Because I think word of mouth, once the first few women successfully navigate the process, they quickly tell their friends.* *- Provider*	Educational materials/toolkits can serve as both a reminder/refresher for clinicians as well as a decisional tool for clients.
**CFIR Construct:** Access to Knowledge & Information **ERIC Recommendation: Involve patients/consumers and family members -** Engage or include patients/consumers and families in the implementation effort	*“Yes, but it’s good that men get involved so they can talk with their partners or girlfriends…But they can motivate them and say, “You know what? We have to be responsible.” Men can’t use an IUD, but women can. So, it’s good that they can talk with girlfriends, partners, or whoever else, right?”* *- Client* *“Honestly, I feel like it could make a good TikTok. Like if it's like — oh, fun fact. Like did you could use this as a emergency... It's just so like easy and fast and it doesn't feel like I’m being lectured, at least on like social media.”* *- Client* *Working with [Instagram] accounts that do empower women, and that make educated resources available to you, and just easy to digest, I think that would be a really helpful way to get this to someone who doesn't really have access to or know to go to WebMD or Planned Parenthood or to Google ‘How will the IUD affect me?’"* *-Client* *“You would want to hear it from your family and friends because those are the people that you kind of trust the most and you can go further into an in-depth conversation and ask them and they would be truthful with you about their experiences more than anyone.”* *- Client*	Education about emergency contraception, including IUD as EC, should include efforts to educate male partners. Education about LNG IUD as emergency contraception should use peer-educators/peer influencers both in-person and through social media to improve visibility.
**CFIR Construct:** Patient Needs & Resources **ERIC Recommendation: Conduct local needs assessment –** Collect and analyze data related to the need for innovation	*“Would they bleed at all? Like you put that [LNG IUD] in, I think a lot of people with emergency contraception like to see that it worked. Would you necessarily bleed after you had it or did you just have to take another pregnancy test or how do you know it worked?”* *-Provider* *So I’ve never counseled for one for using like an LNG IUD but has it ever kind of come up for anybody like how it works for preventing pregnancy? There’s probably a lot of lack of education and probably concern that it's like having an abortion or something...”* *-Provider* *Especially in a domestic violence situation, like say you're trying to get emergency contraception, but if you could also know that if you could get into Planned Parenthood within that five days, you could also get an IUD at the same time, I think that would be really useful.* *-Client*	Information around both the mechanism of action and the expected outcomes of LNG IUD placement for EC in different scenarios are important data needs for future implementation projects.
**CFIR Construct:** Access to Knowledge & Information **ERIC Recommendation: Distribute educational materials -** Distribute educational materials (including guidelines, manuals, and toolkits) in person, by mail, and/or electronically	*[Not identified by participants]*	Distribution considerations are a possibly overlooked component of implementation success.
**CFIR Construct:** Patient Needs & Resources **ERIC Recommendation: Obtain and use patients/consumers and family feedback –** Develop strategies to increase patient/consumer and family feedback on the implementation effort	*[Not identified by participants]*	Channels for receiving and incorporating patient/consumer feedback are possibly an overlooked component of implementation success.

^a^Strategies selected from list created by Powell, B.J., Waltz, T.J., Chinman, M.J. *et al.* A refined compilation of implementation strategies: results from the Expert Recommendations for Implementing Change (ERIC) project. *Implementation Sci* 10, 21 (2015). 10.1186/s13012-015-0209-1

### Intervention characteristics

#### Evidence strength and quality

LNG 52 mg IUDs are not currently FDA-approved as a method of emergency contraception [33, 34]. Some providers participating in the simulations noted that this created some concern around counseling for this use. Providers also would like to see organizations, such as the American College of Obstetrics and Gynecology, adopt formal recommendations for its use as EC. For clients, the focus was on how to interpret the evidence around the effectiveness of the IUD as emergency contraception. Many clients felt that the effectiveness of IUDs as EC should be presented alongside information about the effectiveness of the IUD as a method of contraception, since a person may need both pieces of information to make an informed choice.

#### Complexity

Providers identified several challenges to offering LNG IUD as EC, including the availability of same-day services for insertion. To insert an IUD, the provider typically conducts pregnancy testing. The tests, plus the time needed for counseling and device insertion, often make IUD appointments longer than other contraceptive visits. Providers struggled with the desire to make the LNG IUD as EC available, while also accounting for current low demand for their use as EC and the need for scheduling flexibility to ensure same-day availability. The challenges of LNG IUD as EC largely mirrored existing challenges of providing copper IUD as EC. For clients, there was decisional complexity around the use of the IUD as EC. Aspects such as pain at insertion and the long-term commitment of the method were juxtaposed against the method’s high efficacy at preventing pregnancy. The simulation also identified that counseling around potential contraindications for an IUD could also increase the complexity of offering the LNG IUD as EC.

### Outer setting

#### Patient needs and resources

Providers noted challenges some subpopulations may face in accessing the LNG IUD in an emergency setting. Clients in carceral settings, clients experiencing homelessness, and clients with challenges accessing broader healthcare (e.g., transportation challenges and lack of health insurance coverage) would likely not find this method widely accessible. In-clinic simulations underscored the challenges for clients who were un- or under-insured. Clients also noted that there could be two distinct groups of individuals choosing the LNG IUD as EC. The first group identified would select the LNG IUD because the method was highly effective as EC and the second would choose the LNG IUD as EC because they both needed EC *and* an ongoing method. For those who simply needed the LNG IUD as EC, the issue of how to remove it after the immediate threat of pregnancy had passed was an important consideration, particularly given the high cost of both insertion and removal procedures.

Providers also shared significant concerns about their ability to offer the LNG IUD as EC to adolescents, who they noted are major utilizers of emergency contraception. Providers felt that adolescents, particularly nulliparous adolescents, are more likely to experience high pain levels at insertion and insertion is more likely to be considered difficult.

Clients also discussed cultural considerations around Utah’s largely religious population, noting that partner involvement in contraceptive decision-making may look different when the emergency method ends up being a long-term method.

#### External policy and incentives

In 1983, Utah passed a law preventing clinics receiving state funding from providing care to teens without parental consent. Though this law was ultimately overruled in a court challenge (Planned Parenthood Association of Utah v. Matheson, 1983), its continued existence on the books causes confusion and concern among providers. Providers brought up concerns about their ability to provide care to adolescents in need of EC without parental consent, despite the law being unenforceable. For clients, the over-the-counter availability of oral emergency contraception was seen as an easier option than the process required to obtain an IUD for a similar purpose.

### Inner setting

#### Structural characteristics and compatibility

Simulation trainings demonstrated many challenges to providing IUD as EC services, including the lack of same-day appointments, the availability of clinical staff to support an insertion without a prior appointment, clinic competing priorities for same-day walk-in services (such as COVID vaccines), and stocking challenges of IUDs, which are expensive to purchase without guaranteed use. Providers also noted that scheduling within the required 5-day window for services, particularly if some of those days occur over the weekend, would be a challenge. Similarly, clients noted the difficulty of getting an appointment when desired, given how full most clinics are, a difficulty especially prevalent in low-income clinics. The cost of the IUD was also a main barrier for clients, particularly if it was not fully covered by insurance and the intended use was for a short period of time. This is true for both the copper and LNG IUDs, which can cost more than $1000 if the patient is paying out-of-pocket.

#### Access to knowledge and information

Both providers and clients were unfamiliar with the use of an IUD for EC and demonstrated confusion over the mechanism of action of the LNG IUD as an emergency contraceptive. Clients noted, more broadly, that they were unaware of most other emergency contraceptive options outside of the oral LNG emergency contraceptive (e.g., ulipristal acetate or the copper IUD). Providers who were affiliated with research institutions were more likely to have access to current evidence, such as the effectiveness of LNG IUDs as emergency contraception.

### Characteristics of individuals

#### Knowledge and beliefs about the intervention

A barrier identified during both client and provider focus groups, as well as during simulation training, was a lack of awareness of EC options beyond oral LNG (Plan B). Clients shared that even when they were aware of all their options, they were often confused about where and how to access each of the methods. Providers and clients also lacked understanding about the mechanisms of action for each of the EC methods. Specifically, providers were uncertain about how IUDs work for EC, with some incorrectly believing that IUDs can act as an abortifacient to an established pregnancy, and others unsure if it is appropriate to place an IUD when an individual is at risk of being pregnant but has a negative urine pregnancy test (current evidence [35–37] indicates it is appropriate).

#### Personal attributes

When discussing the use of IUDs (for EC or as ongoing contraception) some providers described IUDs as a method of birth control that is “best” or “right” for their patients. While providers acknowledged that it is ultimately up to the patient to select the method that is best for them, many spoke of the need to persuade patients that the process of inserting IUDs isn’t as bad as they may imagine*.* The view some providers had of IUDs as a universal good stood in stark contrast to experiences shared by some patients. Some patients described the IUD insertion as very painful. Many of those who experienced pain during the insertion wished their providers had been more forthcoming about how painful the insertion could be, and shared recommendations for managing the pain during and following the procedure.

### Recommendations

The CFIR-ERIC Barrier Buster tool identified seven “Level 1” strategies (i.e., where a majority of implementation experts agreed the approach was in their top seven strategies to address a particular CFIR barrier) across four of the nine CFIR constructs identified in our analyses (see Table 3). The seven strategies were (1) identify and prepare champions, (2) conduct educational meetings, (3) develop educational materials, (4) involve patients, (5) conduct local needs assessment, (6) distribute educational materials, and (7) obtain and use patient feedback. A focused implementation package involving these seven strategies should be tested for feasibility and scalability within clinical settings.

**Table 3:** Recommended strategies for implementing use of LNG IUD as EC

CFIR-ERIC Barrier Buster Tool Implementation Strategies Recommended^**a**^	Parallel strategies proposed by study participants	Mapping summary
**CFIR Construct:** Culture **ERIC recommendation: Identify and prepare champions-** Identify and prepare individuals who dedicate themselves to supporting, marketing, and driving through an implementation, overcoming indifference or resistance that the intervention may provoke in an organization	*“I would guess more globally that if ACOG or someone came out saying that it was appropriate and safe that people would feel a lot more easy… they would feel a lot more comfortable doing it simply from a CYA [cover your ass] perspective. I think people are always paranoid that they're going to do something wrong and if it’s supported in guidelines I certainly imagine that more people would feel comfortable with it. I personally think that the data we have is sufficient.”* *- Provider* *I think in our clinic just because sometimes I feel like we’re a little bit more under the microscope. We would probably just really consult with general OB with… I mean we’d probably talk to [Department Chair] and be like, “Listen, are you guys on board with this or not?” And if there’s general consensus that everybody’s on board we would do it but if we didn’t get that sort of nod, we’d probably be like, “Yeah, not doing it.”* *-Provider*	Implementation champions should include both local individuals and those within governing/formal bodies
**CFIR Construct(s):** Access to Knowledge & Information/ Knowledge & Beliefs about the Intervention **ERIC Recommendation: Conduct educational meetings -** Hold meetings targeted toward different stakeholder groups (*e.g.*, providers, administrators, other organizational stakeholders, and community, patient/consumer, and family stakeholders) to teach them about the clinical innovation	*“You also have, other providers have to do CME. If this is something that is introduced in some of the main conferences that people use for their continuing ed, that’d probably help.”* *- Provider* *“Giving informational lectures in churches works well like with mammograms. The church is where women find out where they can go, how, when, and at what cost. Because a lot of women don’t do it because they know or think that if they don’t have insurance, it’s really expensive. Churches are a really good place for that. Or places where are there are a lot of volunteers. For example, I work in the Food Bank. There are a lot of women there. So, the information given there is really good.”* *- Client*	Identifying and using existing venues of knowledge distribution for both clinicians and clients could facilitate educational meetings.
**CFIR Construct:** Access to Knowledge & Information **ERIC Recommendation: Develop educational materials-** Develop and format manuals, toolkits, and other supporting materials in ways that make it easier for stakeholders to learn about the innovation and for clinicians to learn how to deliver the clinical innovation	*“I love the little laminated sheet that I don't know where it comes from. You guys could probably tell me but you guys have all seen. I have the one that tells you all your options and makes it so clear to the patient. Then the one that says — Oops, and then like tells you what the emergency contraception options are. I don't know, if you could replicate those somehow and then make one that includes the LNG IUD, that would be helpful and a good reminder to both patients and providers.”* *-Provider* *Just something along the lines of like the bathroom stall flyers to get hung. It's something that's very simple. I think about the emergency kits that go out with needle exchange and they're full of all sorts of things which this would be an incredible thing to add in there. Just — here are your emergency contraceptive options. By the way, there's a long-term contraceptive option that can be also used as emergency contraception so you could get both things at once but just that is very almost like business card sized and you can stick it anywhere and hand them out by the dozens. Because I think word of mouth, once the first few women successfully navigate the process, they quickly tell their friends.* *-Provider*	Educational materials/toolkits can serve as both a reminder/refresher for clinicians as well as a decisional tool for clients.
**CFIR Construct:** Access to Knowledge & Information **ERIC Recommendation: Involve patients/consumers and family members -** Engage or include patients/consumers and families in the implementation effort	*“Yes, but it’s good that men get involved so they can talk with their partners or girlfriends…But they can motivate them and say, “You now what? We have to be responsible.” Men can’t use an IUD, but women can. So, it’s good that they can talk with girlfriends, partners, or whoever else, right?”* *-Client* *“Honestly, I feel like it could make a good TikTok. Like if it's like — oh, fun fact. Like did you could use this as a emergency... I’d be like, “Hmm, now, I know.” It’s just so like easy and fast and it doesn't feel like I’m being lectured, at least on like social media.”* *-Client* *“I don't get my news from Instagram, but I find that I'm going to Instagram more to kind of digest the news, to kind of get the op-ed piece of my news. So I don't know if it's targeted ads or the sponsorships or what, but working with those accounts that do empower women, and that make educated resources available to you, and just easy to digest, I think that would be a really helpful way to get this to someone who doesn't really have access to or know to go to WebMD or Planned Parenthood or to Google ‘How will the IUD affect me?’”* *-Client* *“You would want to hear it from your family and friends because those are the people that you kind of trust the most and you can go further into an in-depth conversation and ask them and they would be truthful with you about their experiences more than anyone.”*	Education about emergency contraception, including IUD as EC, should include efforts to educate male partners. Education about LNG IUD as emergency contraception should use peer-educators/peer influencers both in-person and through social media to improve visibility.
**CFIR Construct:** Patient Needs & Resources **ERIC Recommendation: Conduct local needs assessment –** Collect and analyze data related to the need for innovation	*“Would they bleed at all? Like you put that [LNG IUD] in, I think a lot of people with emergency contraception like to see that it worked. Would you necessarily bleed after you had it or did you just have to take another pregnancy test or how do you know it worked?”* *-Provider* *So I’ve never counseled for one for using like an LNG IUD but has it ever kind of come up for anybody like how it works for preventing pregnancy? There’s probably a lot of lack of education and probably concern that it's like having an abortion or something...”* *-Provider* *Because I was just thinking about it, especially in a domestic violence situation, like say you're trying to get emergency contraception, but if you could also know that if you could get into Planned Parenthood within that five days, you could also get an IUD at the same time, I think that would be really useful. I think in that situation I would still get the Plan B and still take it, but I'd also schedule an appointment for an IUD, so I'd want to make sure that that was safe, and I wasn't going to take the time and energy to schedule an appointment just to go on and be told that I took Plan B three days ago, I can't do this now.* *-Client*	Information around both the mechanism of action and the expected outcomes of LNG IUD placement for EC in different scenarios are important data needs for future implementation projects.
**CFIR Construct:** Access to Knowledge & Information **ERIC Recommendation: Distribute educational materials -** Distribute educational materials (including guidelines, manuals, and toolkits) in person, by mail, and/or electronically	*[Not identified by participants]*	Distribution considerations are a possibly overlooked component of implementation success.
**CFIR Construct:** Patient Needs & Resources **ERIC Recommendation: Obtain and use patients/consumers and family feedback –** Develop strategies to increase patient/consumer and family feedback on the implementation effort	*[Not identified by participants]*	Channels for receiving and incorporating patient/consumer feedback are possibly an overlooked component of implementation success.

^a^Strategies selected from list created by Powell, B.J., Waltz, T.J., Chinman, M.J. *et al.* A refined compilation of implementation strategies: results from the Expert Recommendations for Implementing Change (ERIC) project. *Implementation Sci* 10, 21 (2015). 10.1186/s13012-015-0209-1

Additional strategies identified by study participants included the development and strengthening both the educational and referral pathways between the pharmacy and clinical care settings, to ensure clients seeking oral EC from a pharmacy were aware of (a) the limitations of oral EC among individuals with higher body mass index (and thus, these individuals may benefit from an IUD as EC, which does not have weight limitations) and (b) that the IUD as EC has higher efficacy for all people, and thus may be an important avenue for people with very high prioritization on not becoming pregnant.

Participants also noted the importance of changing clinical care pathways so that standard contraceptive visits include counseling about and possible provision of EC. Counseling about the LNG IUD as EC during a normal contraceptive visit should also include information about the possibility to have it removed after the immediate threat of pregnancy has passed. Recommendations to ensure provision of LNG IUD as EC was possible in standard clinical settings included the importance of educating the entire medical team (e.g., front desk staff, medical assistants, providers) on how to ensure clients seeking these services could get same-day care, as well as obtaining support from administrative staff on creating openings to provide these services to drop-in clients.

## Discussion

This study assessed potential barriers and facilitators to utilization of the LNG IUD as EC with the aim to develop an implementation intervention. Use of the LNG IUD as EC has many potential benefits to patients. IUDs (both the LNG and copper) are an important option for patients who need highly effective EC, those with higher body mass index for whom oral EC may be less effective [38], and for patients who desire ongoing contraceptive methods. To realize these benefits to patients, interventions aimed at improving accessibility of IUDs as EC will need to address underlying challenges to its implementation. Findings in the CFIR framework demonstrated barriers at the external, internal, and intervention levels, which would require strategies at multiple levels of the health system, including governance, clinical, provider, and patient levels in order to successfully address challenges.

Notably, many barriers identified in our study have also been identified in studies on other methods of emergency contraception [39]. Studies on barriers to use of both ulipristal acetate, a prescription-only oral emergency contraceptive, and the copper IUD as EC, have found issues of knowledge/awareness, cost, and healthcare system barriers to be key components of low utilization [40–42]. Many studies of EC have noted the misperception that these methods result in abortion [39, 43, 44]. Thus, many of the strategies to improve uptake of the LNG IUD as EC are likely also needed to improve uptake of *any* EC method and it is possible that successful interventions could target improving access to EC broadly, rather than simply focusing on LNG IUD as EC. However, some strategies are specific only to the LNG IUD, such as distributing specific evidence around the use of LNG IUD as EC, seeking FDA approval for its use as an EC, and receiving recommendations for its use as EC from organizations that produce clinical guidelines such as ACOG, the Centers for Disease Control and Prevention (CDC), and the World Health Organization (WHO).

Similarly, many of the barriers to use of IUDs as EC are similar to known barriers to IUD use broadly. Cost of intrauterine devices is often prohibitive for patients [45] and the burden of these costs may be further perceived as too high if the device is only used for a short period of time. Addressing IUD insertion pain has been an ongoing challenge for implementors interested in increasing access to these devices [46]. Further, addressing provider bias toward these methods is also a general challenge around intrauterine device use and promotion [47]. Use of the IUD in emergency scenarios likely compounds, rather than diminishes these challenges.

This study sought multiple perspectives in order to fully identify implementation challenges to offering the LNG IUD as EC in clinical settings. Mapping both the CFIR framework and the CFIR-ERIC Barrier tool to participant responses was an effective approach to contextualizing implementation challenges and potential solutions in an intervention planning phase. Potential limitations to our study include the generalizability of our sample, given that all participants live/practice in Utah. As each state and country has different external environments, our findings may not represent the full context or impact of various state/country policies on EC provision and coverage. Intervention designs require local context, as well as expert recommendations, in order to be successful, but individuals interested in implementing this intervention elsewhere could likely map their work onto our findings as a starting point.

## Conclusions

Availability of the levonorgestrel IUD as a new form of EC has potential to benefit many people seeking to prevent pregnancy after unprotected sex who also desire an ongoing method of contraception with a favorable benefit profile. To successfully provide access to this method, implementing teams must support development of robust referral pathways among pharmacy and clinical settings, ensure patients and providers are aware of IUDs as an option for EC, and work with healthcare teams to incorporate IUDs as EC into clinic workflow and standard practice.

## Data Availability

The qualitative data supporting the conclusions of this article is available upon request.

## Abbreviations

CFIR: Consolidated Framework for Implementation Research
EC: Emergency contraception
ERIC: Expert Recommendations for Implementing Change
FDA: Food and Drug Administration
IUD: Intrauterine device
LNG: Levonorgestrel
